# Hand therapy: Inclusive care by South African physiotherapists

**DOI:** 10.4102/sajp.v79i1.1942

**Published:** 2023-11-24

**Authors:** Monique M. Keller

**Affiliations:** 1 Department of Physiotherapy, Faculty of Health Sciences, University of the Witwatersrand, Parktown, South Africa

**Keywords:** physiotherapists, occupational therapists, scope of practice, hand therapy, hand rehabilitation, upper limb rehabilitation, curriculum

## Abstract

Hand therapy for individuals who sustained hand injuries is included in the Health Professions Council of South Africa (HPCSA) physiotherapy scope of practice. The training that physiotherapists receive at the undergraduate level lays the foundation for them to deliver hand therapy or hand rehabilitation according to the International Classification of Functioning, Disability and Health domains in a uniquely South African health service context. Further structured formal and informal postgraduate continued education opportunities may put physiotherapists in the ideal position in a multidisciplinary team to deliver optimal hand therapy. The problem is that the physiotherapist’s role in the multidisciplinary team delivering hand therapy in the past two decades has decreased, leaving room for a lack of health professional services in South Africa’s private sector where physiotherapists are often the first consultation in, for example, sports teams, but more pertinently, in the public and rural areas. The International Federation of Societies for Hand Therapy (IFSHT) practice profile and physiotherapy scope of practice, curriculum and education information assist in achieving the aim of this commentary to position physiotherapists in South Africa as primary health practitioners in delivering hand therapy.

**Clinical implication:**

The effective management of individuals with hand-related conditions and injuries is pertinent to ensure optimal hand function and quality of life. Equal continued formal education opportunities should thus be created for all multidisciplinary team professions at a postgraduate level.

## Introduction

The complexity of the hand’s anatomy allowing tactile feedback, dexterity and precision during fine motor tasks and performing activities of daily living makes the human hand complex and unique (Khan & Sivakumar 2016). Hand injuries are common occurrences globally, ranging from minor cuts and bruises to more severe injuries such as fractures, dislocations and tendon or nerve damage (De Jong et al. 2014). They can result from various causes, including accidents at home, occupational hazards, sports-related injuries and trauma. Cooper and Wietlisbach (2014) indicate that hand therapy is important to ensure optimal hand function post-surgery and during conservative management of hand injuries.

The current problem is that amidst the high burden of trauma leading to hand injuries in South Africa (Naidoo, Govender & Naidoo 2021) and hand injuries’ complexity requiring a multidisciplinary team management approach (Gupta et al. 2013), physiotherapists have not been consistently recognised in the past 20 years as multidisciplinary or interprofessional team members. However, delivering hand therapy is in the physiotherapy scope of practice. With unequal formal postgraduate educational opportunities to develop the knowledge and skills, leaving a gap in health profession service delivery and physiotherapists being rehabilitation experts, a concern exists that do individuals who sustain hand injuries receive optimal rehabilitation in South Africa, especially in traumatic hand injuries that impact on a person’s ability to perform daily activities (Gupta et al. 2023). Therefore, the international practice profile and hand therapy skill set need to be investigated towards transformation in hand therapy service delivery by physiotherapists as part of the interprofessional team in South Africa.

This commentary aims to answer two questions:

- What are the international practice profile and therapeutic skills necessary to be a hand therapist?
- How are physiotherapists trained, past and present, in the sphere of hand injury management in South Africa?

Aspects such as hand injury incidence, mechanism of injuries, direct and indirect costs of managing hand injuries and types of hand injuries need to be considered when recommendations are made on skills and training.

## Incidence and mechanism of hand injuries

The Global Burden of Disease (GBD) obtained from the Institute for Health Metrics and Evaluation (IMHE 2013) reported that injuries sustained accounted for 10.12% of disability adjusted life years, which poses a higher burden than human immunodeficiency virus or acquired immunodeficiency syndrome (HIV or AIDS) (2.84%), malaria (2.68%) and tuberculosis (2%). Although a limitation of the systematic analysis GBD (IMHE 2015) did not specify the injuries, the literature supports hand injuries being among the highest injuries sustained and presented to emergency departments (20%) (De Putter et al. 2012; Polinder et al. 2013).

Hand injuries account for a substantial number of injuries across the globe (Uys, Buchanan & Van Niekerk 2020). Trybus et al. (2006) agree that hand injuries globally constitute the largest proportion of injuries seen in hospitals impacting individuals’ performance in their social responsibilities. When considering hand injuries in developing countries such as Nigeria, the increase in injuries can be related to trauma, violence, road accidents and injuries sustained while participating at work (Ihekire, Salawu & Opadele 2010). Makobore et al. (2015) found that in sub-Saharan Africa, hand injuries were sustained in the workplace (36%), especially while operating machinery (21.3%), at home (28%) and during road accidents (23%). Khan and Sivakumar (2016) state that trauma-related hand injuries are the major cause of dysfunction. When considering hand injuries sustained in KwaZulu-Natal, South Africa, Hardcastle, Samuels and Muckart (2013) reported that because of the extensive trauma in the province, at least 17.8% of all emergency visits are for hand-related injuries.

When considering the types of hand injuries in KwaZulu-Natal, Naidoo et al. (2021) found flexor tendon injuries to occur most often (88%), closely followed by fractures (83%) and extensor tendon injuries (73%). Naidoo et al. (2021) found that occupational therapists who participated in an online survey and focus groups shared that limited resources, poor communication, and late referrals were challenging, impeding effective multidisciplinary management.

## Economic cost of hand injuries

It is not only the functional deficits after hand injuries that are of concern but also the financial implications of hand injuries for the individual, families, employers and government. In a prospective descriptive study, Makobore et al. (2015) aimed to determine the early outcomes and burden of hand injuries in tertiary hospitals in sub-Saharan Africa over a 5-month period. The financial losses identified included treatment delivery, time off work and even loss of employment.

Implementing measures to potentially conserve resources, encompassing direct and indirect costs and medical expenses, can be instrumental, especially in the public health sector of South Africa in the light of the National Health Insurance. Direct costs are associated with the provision of direct care to patients, while indirect costs refer to the financial setbacks experienced by individuals, families and communities because of decreased productivity or the discontinuation of employment, resulting in income loss. This matter is particularly relevant within the context of South African public health, where rehabilitation professionals encounter substantial workloads or face shortages in staffing. The confirmation of resource savings can only be ascertained through future economic evaluations, as illustrated by Robinson et al.’s (2016) systematic review, which confirmed that acute hand and wrist injuries significantly burden individuals and society. The review included a total of 21 articles, with 12 of them focusing on cost-of-illness and seven on health economic evaluations. The findings revealed a median cost per patient of $6951.00 (interquartile range [IQR]: $3357.00 – $22 274.00) for all types of hand and wrist injuries in the cost-of-illness studies, while the health economic evaluations reported a median cost of $8297.00 (IQR: $3858.00 – $33 939.00).

In a systematic review, Siotos et al. (2018) aimed to evaluate the burden caused by hand injuries in low- and middle-income countries and concluded that the healthcare costs are attributed to disability and lack of productivity. The most significant healthcare burden is the direct and indirect costs related to treatment. De Putter et al. (2012) made mention of the peculiar nature of managing hand injuries because of the unique skills required as well as the disability rather than mortality extenuating the burden and costs of healthcare. Cultivating skilled physiotherapists to deliver effective management to individuals with hand injuries or disease is pertinent in South Africa when considering the economic cost, the incidence of hand injuries and the lack of resources.

To address potential past injustices adequately and appropriately with unequal formal postgraduate physiotherapy education opportunities, the international scope of hand therapy practice, the scope of physiotherapy practice pertaining to hand therapy need to be considered.

## The international scope of hand therapy practice

The International Federation of Societies for Hand Therapy (IFSHT) represents 38 national hand therapy organisations, seven associates and 12 corresponding countries with more than 10°000 therapists globally (IFSHT 2010). The IFSHT states that both physical and occupational therapists may specialise in treating patients with upper limb and hand injuries.

The IFSHT (2010) Education committee compiled a hand therapy practice profile outlining:

[*T*]he common elements of the advanced knowledge, clinical practice skill and core competencies that have been used to define the scope of hand therapy practice in different regions of the world, respecting the range of hand therapy practice within the context of wide-ranging diversity of cultural, educational, legal and health care systems worldwide. (p. 1)

De Klerk et al. (2016) agree with the IFSHT that South Africa has a unique context for delivering care in a variety of settings. Coovadia et al. (2009) also refer to the unique South African context where health policies are poorly implemented, and there is shortage of human resources in delivering healthcare services. Patients needing rehabilitation after sustaining hand injuries are seeking care not only in private practices but also in various public health facilities, and at times, home-based care. The public facilities include tertiary hospitals, community health centres and rural settings. The public facilities are often under-resourced, and therapists need to improvise and work in creative ways to administer hand rehabilitation (Coovadia et al. 2009). The application of the IFSHT practice profile that is outlined below is a valuable guide in developing hand therapy educational programmes, especially in a South African context, in which there is a great need for multidisciplinary and interprofessional hand therapy education at undergraduate and postgraduate levels.

The competencies of hand therapists have been established by the Hand Therapy Certification Commission and the following education committees: the Netherlands, the European Federation of Societies for Hand Therapy (EFSHT) and the United Kingdom. International hand certification programmes at a postgraduate level exist in certain countries. The countries that provide international hand therapy certifications and the qualifications that exist are the United States (Certified Hand Therapist), United Kingdom (Accredited Hand Therapists – British Association of Hand Therapists), European countries that are members of the EFSHT (European Certified Hand Therapist) and Australia (Australian Hand Therapy Association member status). In the above certification programmes, physiotherapy and occupational therapy professionals working in the specific country associated with the corresponding certification are invited to apply when meeting the inclusion criteria. In South Africa, there is currently no hand therapy certification and thus no speciality as a hand therapist. There is a divide between physiotherapists and occupational therapists who may manage patients with hand injuries, which may leave the injured individuals in a disadvantaged position. The IFSHT provides a good solution to this problem by recognising the strengths of both physiotherapy and occupational therapy. The solution states that:

[*I*]nterdisciplinary rehabilitation has replaced the traditional boundaries between two professions. It is felt across the IFSHT countries that joint professional teamwork between occupational therapists, physiotherapists and other members of the rehabilitation team is the approach of choice for dealing effectively and efficiently with the complex issues arising in hand rehabilitation. (IFSHT 2010:2)

According to the EFSHT hand therapist profile (EFSHT 2012), hand therapists are occupational therapists and physiotherapists with specialised expertise in managing individuals with upper limb conditions. Upper limb or upper extremity conditions encompass the hand, wrist, elbow, and shoulder girdle. The conditions that may affect the upper extremities encompass a range of health issues, including injury traumas, dysfunctions, illnesses, disorders, diseases, and congenital or acquired deformities. Through extensive continuing education, hands-on experience, and self-directed life-long learning, these professionals have acquired the necessary skills to address such conditions effectively. The primary purpose, thus, of a hand therapist is to facilitate the restoration and preservation of functionality while preventing dysfunction in individuals suffering from disabling upper limb conditions.

To position physiotherapy as part of the interprofessional hand therapy team, information about what skills are required by a hand therapist is needed. According to Dimick et al. (2009) and EFSHT (2009), the specialised therapeutic skills required to be a hand therapist are as follows: training in activities of daily living, assistive or adaptive devices, compression therapy, management of behaviour, electrotherapy, desensitisation, exercise, oedema control, ergonomic modifications, activity, manual therapy and education to the patients and their families. Specialised therapeutics skills required to be a hand therapist are also sensory re-education, prosthetics, splinting or orthotics, scar management, strengthening, wound care and dressings, vocational assessment, work hardening and retraining, thermal modalities and the use of non-standardised and standardised assessment tools. Some aspects of the abovementioned skills pertain more to a specific profession. For example, vocational assessment for occupational therapists and physiotherapists who have not been educated and experienced in it will require upskilling. Similarly with manual therapy and electrical modalities, where physiotherapists are trained at an undergraduate level, occupational therapists pursuing a special interest in hand therapy will require the necessary training.

Having considered the IFSHTs’ view of what constitutes a hand therapist, the focus needs to shift towards the South African physiotherapy scope of practice, undergraduate curriculum where the foundation of knowledge and skill is laid, and postgraduate opportunities or lack of opportunities to pursue expert skills and life-long learning in hand therapy.

## Scope of physiotherapy practice in South Africa

According to the Health Professions Council of South Africa (HPCSA 1976), the scope of physiotherapy will now be presented. Physiotherapy as a profession plays and in future may play a more vital role than is currently seen, in providing hand therapy, functional assessment and orthosis treatment for individuals who have sustained hand injuries. According to the regulations defined by the South African Medical and Dental Council (HPCSA 1976) scope of the profession of physiotherapists, physiotherapy services in orthopaedics, including the specialised branch of hand surgery, are considered supplementary to medicine.

Physiotherapists as first line-practitioners, assess and diagnose hand injuries, such as fractures, ligamentous and soft tissue lesions, joint deformities and diseases. They also address complications from bone infections, amputations and tendon and muscle transplants (HPCSA 1976).

In the context of hand therapy, physiotherapists utilise movement techniques based on physiological principles, to facilitate the restoration of normal physiological processes and functional activities. This involves the scientific use of movement techniques, massage, joint mobilisation and/or manipulation, electrotherapy and other physical and supportive measures. The physiotherapists aim to prevent and treat injuries, diseases and disorders and promote the patient’s independence and overall well-being (HPCSA 1976).

Physiotherapists conduct comprehensive examinations of patients, assess joint range of motion, muscle strength and power, strength, tone, endurance, coordination, balance, posture, functional ability, sensory and motor development and other relevant measurements. Based on the physical diagnosis by the referring medical practitioner and/or the physiotherapy assessment, the physiotherapists select appropriate treatment techniques and supportive devices. These devices include fabricating and adjusting splints, supports, prostheses, aids, appliances and other physiotherapeutic devices for hand function rehabilitation and restoration.

In addition to the physical aspects, physiotherapists also play a role in patient education and advice, that include preventive measures, proper lifting and handling techniques, functional activities, working postures, recreational and sports activities and aids and appliances. This education and advice are tailored to the specific condition diagnosed by the referring medical practitioner and aim to empower patients and their caregivers in managing their hand injuries.

The regulations governing the profession emphasise the collaborative role of physiotherapists, and medical practitioners in delivering comprehensive patient care. Physiotherapists thus employ evidence-based techniques to facilitate hand function recovery and restoration while prioritising patient education and preventive measures.

## Undergraduate physiotherapy curriculum related to hand therapy

The directive of the HPCSA guides the undergraduate curricula in South African universities. The HPCSAs Physiotherapy, Podiatry and Biokinetics (PPB) stipulate, as minimum requirements of training for physiotherapists, that physiotherapy as a profession specialises in the physical assessment and treatment of ‘body structure, organs and systems’ to optimise health (Professional Board for Physiotherapy, Podiatry and Biokinetics 2023).

The IFSHT in the hand therapy practice profile that was amended and adopted in 2010 states that a hand therapist must combine a comprehensive knowledge of anatomy, physiology, pathology, function and biomechanics and conceptual rehabilitation challenges in the upper limb. It is envisaged that through encouraging high standards of care, research and education in the specialised field of hand therapy, as well as promotion, more patients will consult hand therapists and, in turn, increase the likelihood of individuals returning to normal functioning after injury and return to full community participation.

In the undergraduate curriculum at the University of the Witwatersrand the physiotherapy syllabus incorporates theoretical and skills attainment towards a foundation of hand therapy in the courses presented per year of study, which now follow. First-year courses include an introduction to physiotherapy, physics, basic principles of group and individual psychology, introduction to psychology and human behavioural sciences. Second-year courses are anatomy for physiotherapy and occupational therapy students, physiology and biochemistry and physiotherapy I. The third year of study includes physiotherapy II, clinical physiotherapy I and general medicine and surgery, which consists of the pathology, surgical and medical management of hand conditions (University of the Witwatersrand 2023). The final year (fourth-year) courses include management for therapists, theoretical content in the physiotherapy and rehabilitation III courses and practical skill acquisition in the clinical physiotherapy II course. The undergraduate physiotherapy courses provide a foundation for physiotherapists with further postgraduate hand therapy education to become skilled and life-long learners underpinning the IFSHT hand therapy practice profile.

## Postgraduate education in hand therapy

South African postgraduate education in hand therapy is provided by informal weekend or midweek evening lectures presented by experts on the topic and are hosted by the South African Society of Hand Therapy. Previously, for South Africans, the Postgraduate Diploma in Hand Therapy provided formal training by providing theoretical knowledge and practical skills. Before and including 2002, the diploma was offered to physiotherapists and occupational therapists. Anecdotally, the diploma curriculum difference between professions occurred when physiotherapists were exposed to more splinting or orthosis training and occupational therapists to electrotherapy modalities. Albeit the split in training, the rest of the curriculum was similar for both professions. Since 2004, no physiotherapists have been allowed to enrol in the diploma by the diploma coordination committee, with the reason provided for the exclusion being that ‘physiotherapists are not allowed’. Hence, this background of the postgraduate diploma explains where the opportunities for physiotherapists to gain hand therapy knowledge and skill were lost.

Evidence of physiotherapists enrolling in the Postgraduate Diploma in Hand Therapy can be found in the HPCSAs *Health Professions Act 56 of 1974* ‘Rules relating to the registration by physiotherapists of additional qualifications’. It was published under the ‘Board Notice 72 in Government Gazette 30050 of 13 July 2007’. In the act, under the University of Pretoria, the Postgraduate Diploma in Hand Therapy is documented as an additional qualification physiotherapist could attain. The only other programme offered to both professions was the Masters in Hand Rehabilitation offered at the University of KwaZulu-Natal, which since its last intake in 2014, has closed.

When considering the undergraduate physiotherapy curriculum, a foundation is laid. However, according to the IFSHT, it takes 5 years of further education to be a skilled hand therapist. It is of concern that in South Africa, with its health service delivery and limited resources, as mentioned above, physiotherapists with an undergraduate rehabilitation foundation and the scope of practice to deliver hand therapy are not recognised and included in past and present formal postgraduate education. Often, community service physiotherapists and occupational therapists, with their undergraduate training foundation in hand therapy, provide hand therapy in public, rural and home environments. Considering the limited resources in the public health context and poorly implemented health policies, interprofessional education and practice are required to rehabilitate individuals who sustain hand injuries adequately.

## Recommendations

Not only is the author, a physiotherapist, not in a position to comment on the South African occupational therapy undergraduate curricula but also it is not an objective of the manuscript. However, as a reflection on the past Diploma in Hand Therapy programme, to become a skilled therapist with a special interest in hand therapy requires education at a postgraduate level for both physiotherapists and occupational therapists. No hand therapy specialisation currently exists in South Africa, as compared with international specialisations, and even when a certification exists in the future, the therapists may pursue private practice, leaving the public sector hand therapy delivery to generalist therapists. It is therefore recommended that to develop holistic hand therapists working together in an interdisciplinary team, drawing on the strengths of both occupations, is required in South Africa. It is therefore recommended that future studies investigate any existing multidisciplinary and interprofessional hand therapy practice and the outcomes in the private and public context in South Africa. A recommendation is also to determine whether South African universities include knowledge related to hand therapy in the curriculum and investigate the constructive alignment of the teaching pedagogies. Future undergraduate training should be presented to both professions in combined sessions to build an appreciation of the interprofessional team. Finally, future postgraduate certifications and diplomas in South Africa should be instituted for physiotherapists and occupational therapists based on the IFSHT standard of practice.

## Conclusion

No current hand therapist certification exists in South Africa, and therefore patients presenting with injuries or diseases affecting the hand and upper limb are managed by generalist physiotherapists and occupational therapists. A need for interprofessional hand therapy practice and continued education is needed to optimally deliver the services needed to reach all patients in the vast and diverse health service areas in South Africa.
